# Unbiased Lipidomic Profiling of Triple-Negative Breast Cancer Tissues Reveals the Association of Sphingomyelin Levels with Patient Disease-Free Survival

**DOI:** 10.3390/metabo8030041

**Published:** 2018-07-13

**Authors:** Preeti Purwaha, Franklin Gu, Danthasinghe Waduge Badrajee Piyarathna, Theckelnaycke Rajendiran, Anindita Ravindran, Angela R. Omilian, Sao Jiralerspong, Gokul Das, Carl Morrison, Christine Ambrosone, Cristian Coarfa, Nagireddy Putluri, Arun Sreekumar

**Affiliations:** 1 Alkek Center for Molecular Discovery and Department of Molecular and Cell Biology, Baylor College of Medicine, Houston, TX 77030, USA; purwaha@gmail.com (P.P.); fgu@bcm.edu (F.G.); DanthasingheWaduge.Piyarathna@bcm.edu (D.W.B.P.); Anindita.Ravindran@bcm.edu (A.R.); jiralers@bcm.edu (S.J.); coarfa@bcm.edu (C.C.); putluri@bcm.edu (N.P.); 2 Verna and Mars McLean Department of Biochemistry and Molecular Biology, Baylor College of Medicine, Houston, TX 77030, USA; 3 Michigan Center for Translational Pathology, University of Michigan, Ann Arbor, MI 48109, USA; tmraj@med.umich.edu; 4 Roswell Park Comprehensive Cancer Center, Buffalo, NY 14263, USA; Angela.Omilian@RoswellPark.org (A.R.O.); Gokul.Das@RoswellPark.org (G.D.); carl.morrison@omniseq.com (C.M.); Christine.Ambrosone@RoswellPark.org (C.A.)

**Keywords:** lipidomics, sphingomyelin, sphingolipid, triple-negative breast cancer

## Abstract

The reprogramming of lipid metabolism is a hallmark of many cancers that has been shown to promote breast cancer progression. While several lipid signatures associated with breast cancer aggressiveness have been identified, a comprehensive lipidomic analysis specifically targeting the triple-negative subtype of breast cancer (TNBC) may be required to identify novel biomarkers and therapeutic targets for this most aggressive subtype of breast cancer that still lacks effective therapies. In this current study, our global LC-MS-based lipidomics platform was able to measure 684 named lipids across 15 lipid classes in 70 TNBC tumors. Multivariate survival analysis found that higher levels of sphingomyelins were significantly associated with better disease-free survival in TNBC patients. Furthermore, analysis of publicly available gene expression datasets identified that decreased production of ceramides and increased accumulation of sphingoid base intermediates by metabolic enzymes were associated with better survival outcomes in TNBC patients. Our LC-MS lipidomics profiling of TNBC tumors has, for the first time, identified sphingomyelins as a potential prognostic marker and implicated enzymes involved in sphingolipid metabolism as candidate therapeutic targets that warrant further investigation.

## 1. Introduction

Triple-negative breast cancer (TNBC) comprises a heterogeneous subgroup of breast tumors characterized by an aggressive clinical course and increased likelihood of recurrence [1,2]. Unlike tumors expressing hormone receptors or HER2, TNBC is not responsive to hormone therapy or treatments directed to HER2, emphasizing the need for new targeted treatments for TNBC [3,4]. Clinical outcomes for TNBC patients are associated with race and ethnicity, with non-Hispanic African-American women being more likely to be diagnosed with TNBC and to have poorer survival outcomes than European-American women, even after adjustment for socioeconomic status [5,6,7].

Altered lipid metabolism is a common characteristic of several cancer types, including breast cancer [8,9,10,11]. In particular, sphingolipids are a class of membrane lipids implicated in breast cancer progression [12,13,14]. In addition to their role in maintaining membrane structure, sphingolipids such as ceramide and sphingosine-1-phosphate (S1P) serve as messengers in lipid signaling in cancer cells [15,16,17,18,19,20,21]. Increased ceramide production by cancer cells following chemotherapy treatment has been shown to induce apoptosis and ceramide analogs have themselves shown promise as potential cancer treatments [22,23]. S1P production by cancer cells also simulates angiogenesis through lipid signaling to the tumor microenvironment [24,25]. It has also been shown that sphingoid bases such as sphinganine and sphingosine, which serve as metabolic intermediates in the synthesis of ceramide and S1P, can also induce apoptosis in cancer cells [26]. Interestingly, despite the traditional pro-apoptotic role of ceramides in cancer cells, increased synthesis of certain ceramides in breast cancer tissues has been reported to be associated with cancer progression, suggesting that further investigation is required to investigate the complexity of sphingolipid signaling in cancer cells [27]. In particular, while hormone receptor status has been linked to changes in phospholipid and sphingolipid content in breast cancer cells, the specific changes in lipid metabolism occurring within the TNBC subtype that lead to disease progression are comparatively less well understood [28].

The aim of the current study was to perform unbiased lipidomic screening of TNBC tissues and to identify lipidomic signatures associated with clinical outcomes in TNBC patients. High-resolution liquid-chromatography-mass spectrometry (LC-MS) was used to characterize global lipidomic profiles in 70 invasive breast tumor samples. Our lipidomics platform identified 15 endogenous lipid classes in this cohort of TNBC patients, which were further analyzed for association with patient outcomes within the TNBC group. Decreased levels of sphingomyelins were found to be associated with lower disease-free survival in TNBC patients. Consistent with this metabolic data, independent gene expression analysis of a publicly available TNBC dataset found that increased expression of enzymes involved in the major pathways of ceramide synthesis, including the sphingomyelinase pathway, are associated with decreased disease-free survival. Together, these results show that altered sphingolipid metabolism is associated with disease progression in TNBC. These findings provide a basis to further explore the role of sphingolipid metabolism in TNBC, which in turn could lead to new therapeutic targets and prognostic markers for TNBC.

## 2. Results

**Figure 1:**
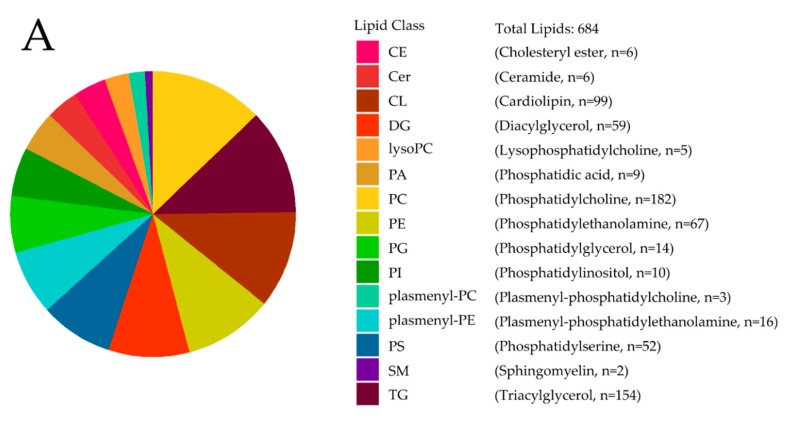

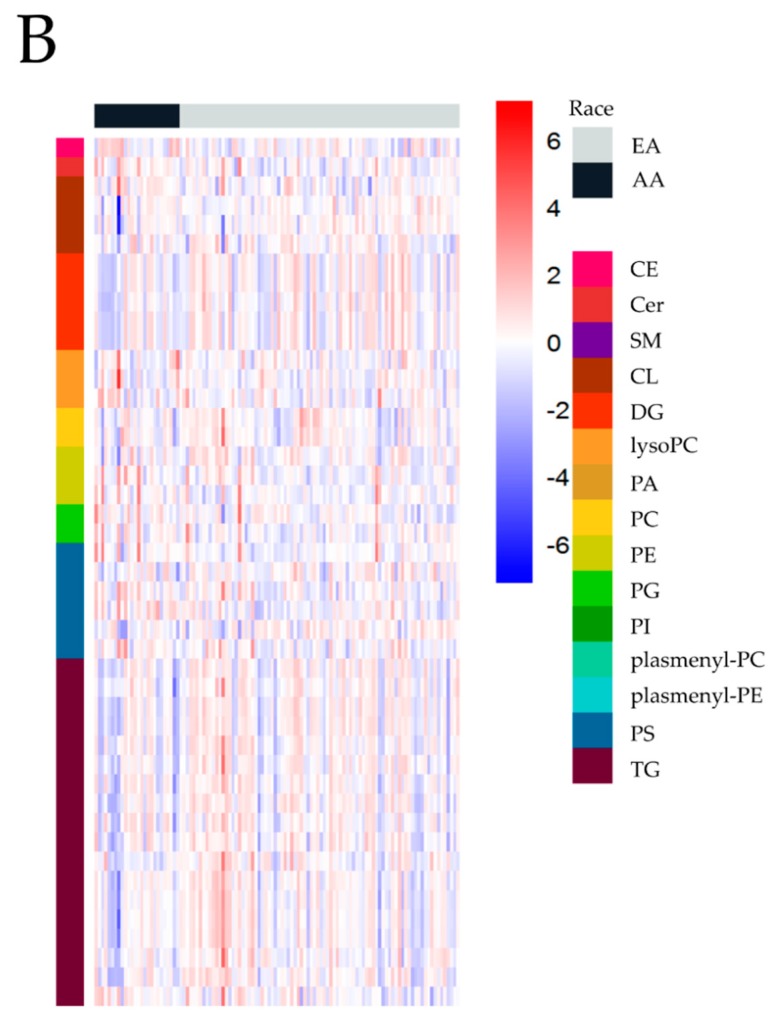
Lipidomic profiling of triple-negative subtype of breast cancer (TNBC) tumors reveals that changes in lipid metabolism are associated with tumor site and racial ancestry. (**A**) Pie chart depiction of the 684 lipids measured and lipid class representation. (**B**) Heatmap of differential lipids between African-American (AA) and European-American (EA) patients in TNBC tumor tissue (false discovery rate (FDR)-adjusted *p* value < 0.25).

High-resolution LC-MS measured a total of 684 named lipids (393 in positive ionization mode; 291 in negative ionization mode) in 70 invasive breast tumor samples mostly belonging to the TNBC subtype (Table 1). Detected lipids fell into 15 endogenous lipid classes (Figure 1A). Self-reported ancestry in the clinical data allowed for a comparison of the lipidomic profiles of African-American (AA) and European-American (EA) TNBC. Intriguingly, in AA vs. EA, among altered lipids (52 lipids in 10 lipid classes; false discovery rate (FDR) *p* value < 0.25) certain phospholipids (PS, PG) increased while most glycerolipids (DG, TG) were reduced (Figure 1B, Supplementary Data 2).

To understand the role of lipid metabolism in TNBC progression, lipid class scores were calculated by summation of the abundances of individual lipids in each class. Multivariate Cox proportional hazards regression including tumor stage was used to determine which lipid classes were the most strongly associated with disease-free survival. Due to sample size limitations in the patient cohort, only Grade III tumors, histologically classified as invasive ductal carcinoma (IDC), were included (*n* = 45). Of the 15 lipid classes measured in our sample cohort with a median follow-up time of 37.5 months, only the class of sphingoid bases was prognostic for disease-free survival in TNBC (Table 2). Further separation of detected sphingoid bases into the classes of ceramides and sphingomyelins revealed that only sphingomyelins were associated with better patient prognosis compared to other lipid classes (Table 3).

**Figure 2:**
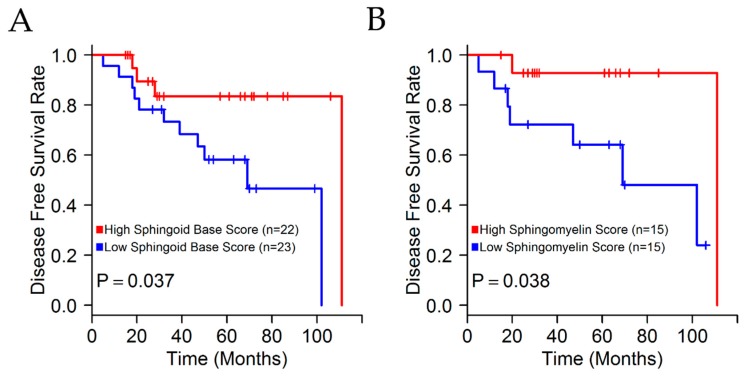
Elevated sphingomyelin levels in primary TNBC samples are prognostic for improved patient survival. (**A**) Kaplan-Meier curves of TNBC patients stratified based on median sphingoid base abundance (log rank test). (**B**) Kaplan-Meier curves of TNBC patients stratified based on highest vs. lowest tertile of sphingomyelin abundance (log rank test).

**Figure A1:**
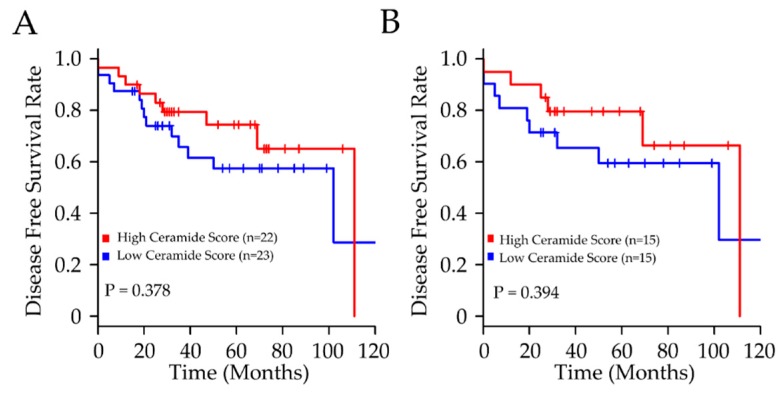
Elevated ceramide levels in primary TNBC samples are not prognostic for patient disease-free survival. Kaplan-Meier curves of TNBC patients stratified based on (**A**) median ceramide abundance or (**B**) highest vs. lowest tertile of sphingomyelin abundance (log rank test).

The association of higher levels of sphingoid bases with better prognostic outcome for TNBC patients was determined using Kaplan-Meier survival plots, with TNBC patients divided into high or low sphingoid base groups based on whether the sphingoid base class score was above or below the median of TNBC samples (Figure 2A). In addition, patient survival was also improved in the group with the highest tertile of sphingomyelin score as compared to the lowest tertile group (Figure 2B). However, similar survival analyses of ceramide levels did not show any prognostic significance (Figure A1, in Appendix A).

**Figure 3:**
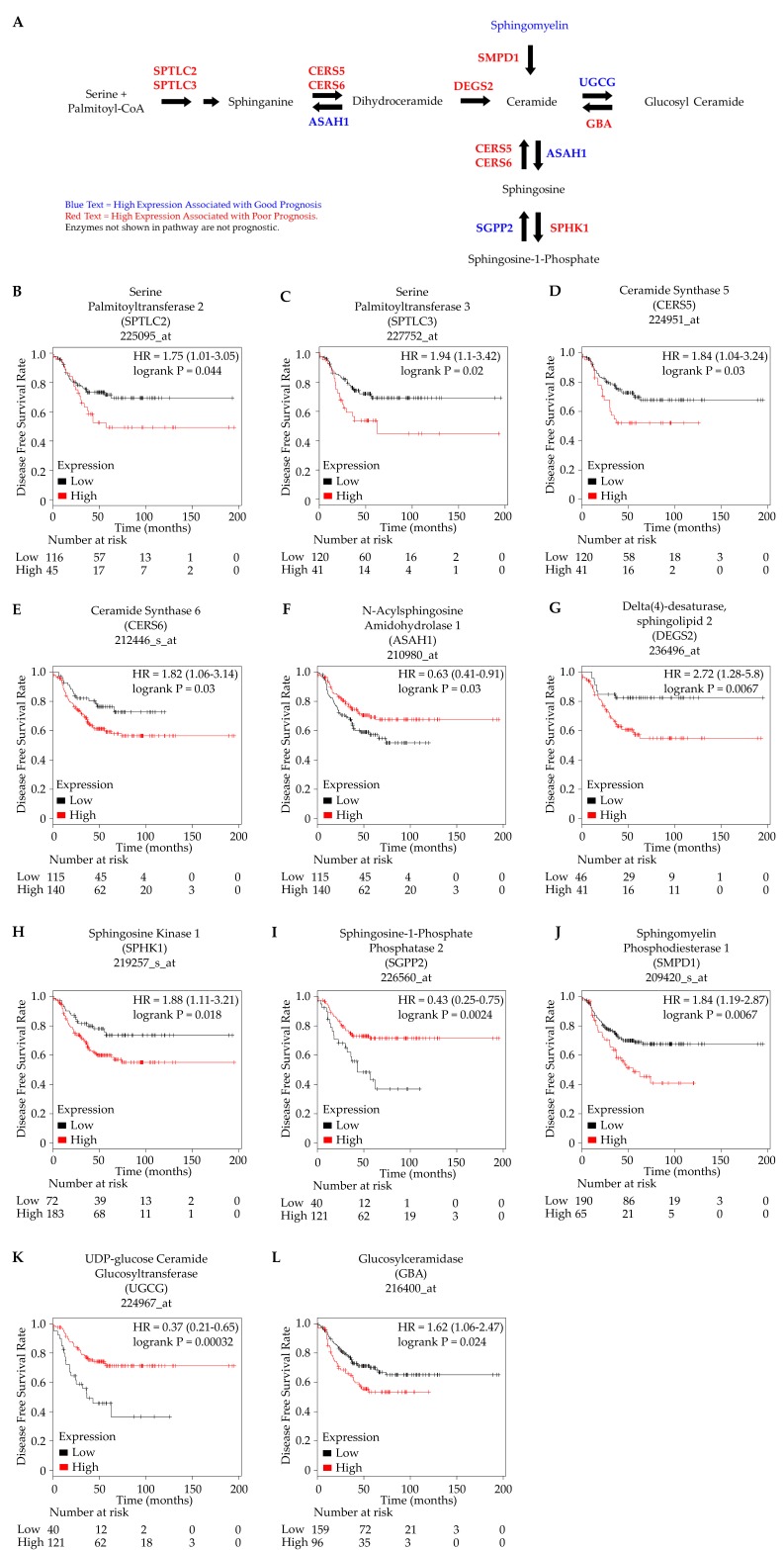
Survival analysis of TNBC patients based on the gene expression of enzymes involved in sphingolipid metabolism. (**A**) Pathway diagram depicting the association of altered enzyme expression involved in sphingolipid metabolism and its effect on disease-free survival outcome. (**B**) SPTLC2, (**C**) SPTLC3, (**D**,**E**) CERS5, CERS6, (**F**) ASAH1, (**G**) DEGS2, (**H**) SPHK1, (**I**) SGPP2, (**J**) SMPD1, (**K**) UGCG, and (**L**) GBA gene expression were determined to be associated with disease-free survival.

To obtain additional insights into sphingolipid metabolism in TNBC, we investigated the expression of genes involved in sphingoid base metabolism using gene expression data publicly available through the online tool KM Plotter (Supplementary Data 3) [29]. Consistent with our lipidomic results, disease-free survival analysis in TNBC patients using optimized cutoff values defined by KM Plotter showed that the increased expression of an enzyme involved in ceramide synthesis through the hydrolysis of sphingomyelin (SMPD1) was associated with poor disease-free survival. In addition, the high expression of metabolic genes involved in de novo ceramide synthesis (SPTLC2, SPTLC3, CERS5, CERS6) was significantly associated with worse disease-free survival. In contrast, the increased expression of enzymes involved in the conversion of ceramides to other sphingolipids (ASAH1, UGCG) was associated with better disease-free survival in TNBC patients. Finally, an enzyme that converts sphingosine to sphingosine-1-phosphate (SPHK2) was significantly associated with poor disease-free survival, while an enzyme catalyzing the reverse reaction was associated with better survival (SGPP2; Figure 3). These results corroborate our findings that tissue levels of sphingolipids, in particular sphingomyelin, are linked to disease progression in TNBC patients.

## 3. Discussion

Several studies have shown that lipids can serve as biomarkers to differentiate breast tumor from normal tissues and that changes in lipid levels are associated with disease progression and hormone receptor status in breast tumors [30]. In addition, a molecular basis for racial disparity in TNBC exists, with biological changes such as increased inflammation and altered gene expression observed in AA TNBC patients who also tend to have worse outcomes [31,32]. Overall, our platform was able to identify an increase in phospholipids and a reduction in glycerolipids in AA compared to EA TNBC tumors. Notably, some of these phospholipid classes (PS) were previously found to be increased in metastatic breast cancer cells relative to less-metastatic breast cancer cells [28]. Together, these results indicate that the accumulation of phospholipids and the depletion of glycerolipids may represent a biological basis for racial disparity and disease progression in TNBC, and should be further investigated.

Sphingolipids such as S1P and ceramides are known to have regulatory roles in controlling tumor proliferation, migration, and angiogenesis [15,16,25]. While ceramides and S1P have traditionally been assigned opposing roles in promoting and inhibiting apoptosis in cancer cells, respectively, it is now recognized that the role of ceramides in cancer progression is much more complex [17,33,34]. Ceramides containing different fatty acid chain lengths have been shown to either oppose or promote apoptosis in head and neck squamous cell carcinoma xenograft tumors, and the inhibition of ceramide synthesis through sphingomyelin hydrolysis reduced tumor growth through the enhancement of antitumor immunity in non-small cell lung carcinoma [35,36]. In breast cancer, the levels of both ceramide and sphingomyelins have been found to be elevated in breast tumors, although ceramide levels have also been shown to be inversely associated with cancer aggressiveness [12,37]. Indeed, our results show that sphingomyelin accumulation as well as decreased sphingomyelinase expression (SMPD1) are both associated with better prognosis in TNBC patients, indicating that ceramide synthesis through sphingomyelin hydrolysis may play a role in promoting TNBC progression, similar to what has been reported in other cancers [36].

The activation of the de novo ceramide synthesis pathway that generates ceramides from serine and palmitoyl-CoA has been shown to mediate apoptosis in response to anticancer agents [38,39,40,41]. Sphinganine is a sphingoid base intermediate produced during de novo ceramide synthesis and ceramides can be cleaved to form sphingosine. These sphingoid bases (sphingosine and sphinganine) have previously been evaluated for their chemotherapeutic and chemopreventive potential, using a novel cell culture system comprising of normal human breast epithelial cells (HBEC) collected from breast tissues of healthy women during reduction mammoplasty procedures [26]. Both sphinganine and sphingosine were found to inhibit the growth and induced apoptosis of transformed tumorigenic Type I HBEC and arrested the cell cycle at G(2)/M, suggesting that sphingoid bases may serve as chemotherapeutic as well as chemopreventive agents by preferentially inhibiting cancer cells and eliminating the stem cells from which most breast cancer cells arise [26]. Consistent with this, our analysis found that the increased expression of enzymes involved in the production of sphinganine and sphingosine (ASAH1, SGPP2) were associated with better disease-free survival in TNBC patients, suggesting that besides ceramide production, the accumulation of sphingoid base intermediates can also inhibit cancer progression. It has also been reported that de novo ceramide synthesis is activated in breast tumors and associated with worse prognosis [37]. This study found that the conversion of ceramides to S1P and an elevated S1P to ceramide ratio could potentially explain why increased ceramide synthesis was associated with worse prognosis [37]. Our gene expression analysis also showed that increased de novo ceramide synthesis enzymes (SPTLC2, SPTLC3, CERS5, CERS6) and sphingosine kinase 1 (SPHK1) expression were associated with worse patient outcome, although our lipidomics method was not able to measure S1P levels directly.

Ceramides can also be converted into glucosylceramide by the enzyme ceramide glucosyltransferase (UGCG) to form complex sphingolipids. Consistent with the pro-apoptotic role of ceramide, the increased expression of UGCG in colon cancer cells was found to promote multidrug resistance through the depletion of ceramide levels [42]. Interestingly, our analysis found that in TNBC patients, increased UGCG was instead associated with better disease-free survival. Furthermore, the expression of a salvage enzyme that recovers ceramide from glucosylceramide (GBA) was shown to be associated with worse patient outcome, suggesting that the activation of the ceramide salvage pathway may promote TNBC progression rather than inhibit it.

In the current study, high-resolution mass spectrometry-based unbiased lipidomic analysis of Grade III TNBC tissues showed that increased levels of sphingomyelins were associated with better disease-free survival outcome. In addition, in silico gene expression analysis of TNBC patient data showed that the increased expression of sphingomyelinase (SMPD1) was associated with worse prognosis. Together, these results indicate that ceramide synthesis through the hydrolysis of sphingomyelin promotes TNBC progression. We also showed that the increased expression of de novo ceramide synthesis (SPTLC2, SPTLC3, CERS5, CERS6) and ceramide salvage (GBA) enzymes were associated with worse prognosis. Furthermore, the increased expression of enzymes that metabolize ceramide to other sphingolipids (ASAH1, UGCG) were associated with better prognosis. Collectively, these findings indicate that the ability to synthesize ceramide through any of the three biosynthetic pathways promotes TNBC progression. Although the mechanism through which ceramide accumulation promotes TNBC progression remains unknown, our analysis indicates that the production of S1P from ceramides (SPHK1) may play a role. Further studies using a targeted lipidomics approach for ceramide metabolism would be required to determine the metabolic pathways contributing most to the ceramide levels in TNBC tumors. Additional studies that perturb the activity of sphingomyelinase and other prognostic ceramide metabolic enzymes identified in this study should be conducted to determine how each metabolic pathway contributes to TNBC progression.

## 4. Materials and Methods

**Figure A2:**
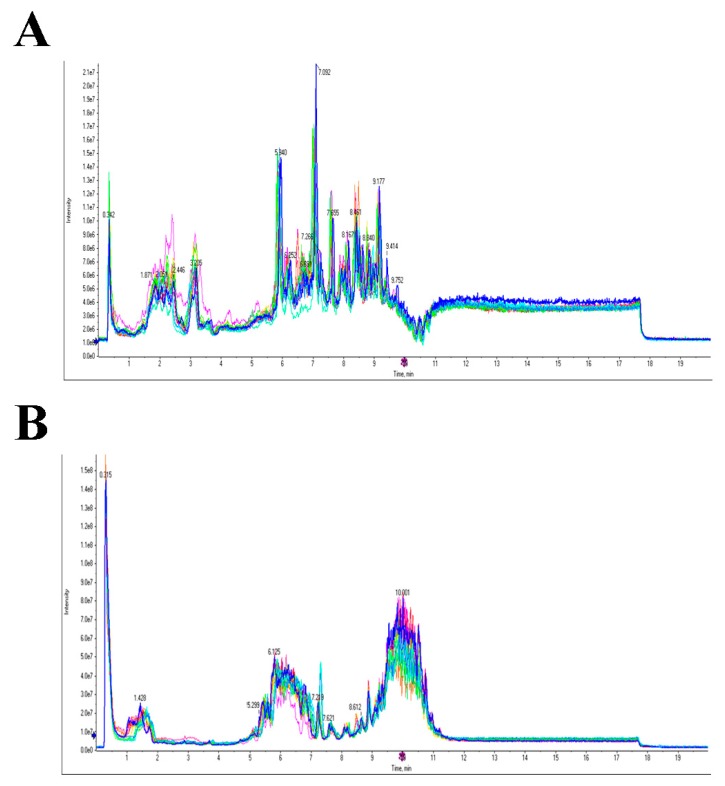
Overlay of the total ion chromatograms for the pooled tissue samples used as quality controls acquired on different days, showing the reproducibility of data acquisition on different days in (**A**) negative ESI ionization mode and (**B**) positive ESI ionization mode.

Reagents, Internal Standards, and Quality Controls: HPLC grade acetonitrile and dichloromethane were purchased from Sigma (St. Louis, MO, USA). LC/MS grade isopropanol, water, and methanol were purchased from Fisher Scientific (New Jersey, NJ, USA) and from J.T. Baker (Radnor, PA, USA). MS grade lipid internal standards were purchased from Avanti Polar Lipids (Alabaster, AL, USA). Internal standard stock solutions were prepared by weighing an exact amount of the lipid internal standard in chloroform/methanol/H_2_O, resulting in a concentration of 1 mg/mL, and they were stored at −20 °C. The stock solutions were then further diluted to result in a final concentration of 100 pmol/µL by mixing an appropriate volume of the internal standard stock [43]. Two different quality controls were used to monitor the variation in sample preparation and instrument performance. To monitor the day to day variability in MS performance, we used a 10=μL injection of matrix-free internal standard mix reconstituted to a final volume of 100 μL in buffer B (5% water, 85% isopropanol, 10% acetonitrile in 10 mM NH_4_OAc). To monitor the variability in the lipid extraction process, we prepared a standard pool of tissue samples that was analyzed every day at the beginning and at the end of the queue of samples. The reproducibility of the standard pool of tissue samples was determined by an overlay of the total ion chromatograms acquired on different days, as shown in Figure A2 (in Appendix A).

Data acquisition through LC/MS Analysis: Twenty milligrams of frozen tissue was accurately weighed and lipid extraction was carried out as described previously [43]. A Shimadzu CTO-20A Nexera X2 UHPLC systems equipped with a degasser, binary pump, temperature-controlled autosampler, and a column oven was used for efficient chromatographic separation. Reverse-phase chromatography was used to separate the lipidome. Ten microliters of sample was injected to a 1.8 μm particle 50 × 2.1 mm Acquity HSS UPLC T3 column (Waters, Milford, MA, USA) heated to 55 °C. Mobile phase A was acetonitrile/water (40:60, *v*/*v*) with 10 mM ammonium acetate and Mobile phase B was acetonitrile/water/isopropanol (10:5:85 *v*/*v*) with 10 mM ammonium acetate. A linear gradient was used over a 20-min total run time, with 60% mobile phase A and 40% mobile phase gradient for the first 10 min. Then the gradient was ramped in a linear fashion to 100% solvent B and maintained for 7 min, after which it was switched back to 60% solvent B and 40% solvent A for 3 min. The flow rate used was 0.4 mL/min. Data was acquired in both positive and negative ionization modes for each sample using a Triple TOF 5600 equipped with a Turbo V^TM^ ion source (AB Sciex, Concord, ON, Canada). The column effluent was directed to the electrospray ionization source for MS analysis. Further details of data acquisitions and processing can be accessed from a previous publication [43].

Statistical analysis: Following data acquisition, duplicated samples and metabolites of non-human origin were removed. Missing values for each lipid were imputed on a per-method basis using the nearest neighbor algorithm with k set to the square root of the number of lipids detected using the R package *VIM* [44]. Imputed data were then log2 transformed. The coefficient of variation (CV) was calculated for each metabolite and metabolites with CV > 20% were removed, followed by median centering. Positive and negative ionization mode data were then combined for further statistical analysis. Lipid class scores were calculated by summing the values for individual lipids in each class corresponding to the LIPID MAPS Lipid Classification System [45]. Calculated lipid class scores in combination with clinical tumor stage were used in a multivariate Cox proportional hazard model to identify risk factors for disease-specific survival. Differential analysis of lipid levels and survival analysis were performed using the R packages limma and survival, respectively [46]. False discovery rate (FDR) correction within the *limma* package was performed using the Benjamini-Hochberg procedure for the determination of differential lipids between patients of different race. Figures were generated using the R packages ggplot2 and pheatmap [47,48].

Sample acquisition: All human samples and related clinical data were obtained in a de-identified manner from Roswell Park Comprehensive Cancer Center. The entire study was conducted with approval from the Institutional Review Boards of Roswell Park Comprehensive Cancer Center and Baylor College of Medicine. All mass spectrometry data (both raw and normalized data) is included in the Supplementary Materials.

## Figures and Tables

**Table 1:** Clinical parameters of breast tumor samples.

Clinical Variable	Breast Tumor Samples (%) (*n* = 70)
**Receptor Status, *n* (%)**	
Triple-Negative	70 (100)
ER+	0 (0)
**Race, *n* (%)**	
African-American	14 (20)
European-American	53 (75.7)
Other	3 (4.3)
**Histological Type, *n* (%)**	
Ductal	57 (81.4)
Other	13 (18.6)
**Grade, *n* (%)**	
II	6 (8.6)
III	63 (90)
Other	1 (1.4)
**AJCC Stage, *n* (%)**	
1	17 (24.3)
2	33 (47.1)
3	14 (20)
4	2 (2.9)
Unknown	4 (5.7)
**Sample Site, *n* (%)**	
Primary	66 (94.3)
Metastatic	4 (5.7)
**Clinical Follow-Up (months)**	
Mean	45.6
Median	35
Standard Deviation	32.4

**Table 2:** Multivariate Cox regression analysis to identify prognostic factors in primary TNBC tumors.

Factors	Disease-Free Survival		
	HR	95% CI	*p* Value
**Stage (I/II vs. III)**	0.70	0.11–4.5	0.71
**CE**	1.22	0.85–1.75	0.27
**DG**	1.00	0.98–1.02	0.67
**LysoPC**	1.07	0.88–1.32	0.45
**PA**	0.86	0.65–1.15	0.32
**PC**	0.99	0.97–1.02	0.84
**Plasmenyl-PC**	1.19	0.62–2.3	0.59
**Plasmenyl-PE**	0.86	0.7–1.06	0.18
**TG**	1.00	0.98–1.02	0.77
**CL**	1.04	0.98–1.09	0.12
**SB**	0.77	0.6–0.98	0.03
**PE**	0.98	0.92–1.04	0.55
**PG**	0.93	0.8–1.07	0.33
**PI**	1.17	0.98–1.4	0.06
**PS**	1.01	0.95–1.08	0.55

**Table 3:** Multivariate Cox regression analysis to identify prognostic factors in primary TNBC tumors in sphingoid base classes.

Factors	Disease-Free Survival		
	HR	95% CI	*p* Value
**Stage (I/II vs. III)**	1.02	0.12–8.13	0.98
**CE**	1.56	0.91–2.66	0.1
**DG**	0.99	0.96–1.01	0.47
**LysoPC**	1.11	0.88–1.4	0.37
**PA**	0.86	0.61–1.22	0.41
**PC**	0.98	0.95–1.01	0.36
**Plasmenyl-PC**	1.2	0.53–2.72	0.65
**Plasmenyl-PE**	0.89	0.71–1.11	0.31
**TG**	0.99	0.97–1.01	0.81
**CL**	1.02	0.97–1.08	0.32
**Cer**	1.09	0.76–1.55	0.61
**SM**	0.37	0.17–0.77	0.008
**PE**	0.98	0.91–1.05	0.59
**PG**	0.92	0.78–1.09	0.35
**PI**	1.18	0.97–1.44	0.08
**PS**	1.04	0.97–1.11	0.25

## Supplementary Materials

The following are available online at http://www.mdpi.com/2218-1989/8/3/41/s1, Supplementary Data 1: Raw and normalized lipidomic data generated from breast tissues including 70 breast tumors during this study, Supplementary Data 2: Table of differential analysis to determine lipids changing between patients of different racial ancestry, Supplementary Data 3. Table of sphingolipid metabolic genes analyzed for association with patient disease-specific survival.
