# Supplementary material for: Unbiased Lipidomic Profiling of Triple-Negative Breast Cancer Tissues Reveals the Association of Sphingomyelin Levels with Patient Disease-Free Survival
Source: Metabolites. 2018 Jul 13;8(3):41. doi: 10.3390/metabo8030041 (PMC6161031; doi:10.3390/metabo8030041)
Supplement: Supplementary file 1 [file metabolites-08-00041-s001.zip › Supplementary/SupplementaryCaptions.docx]

**Supplementary Data 1.** Raw and normalized lipidomic data generated from breast tissues including 70 breast tumors during this study.

**Supplementary Data 2.** Table of differential analysis to determine lipids changing between patients of different racial ancestry.

**Supplementary Data 3.** Table of sphingolipid metabolic genes analyzed for association with patient disease-specific survival.
